# Clinical Characterization of Invasive Meningococcal Disease Epidemic, Uzbekistan, 2026 

**DOI:** 10.3201/eid3210.261144

**Published:** 2026-10

**Authors:** Bakhodir B. Rakhimov, Laziz N. Tuychiev, Nilufar T. Khamzayeva, Nigora U. Tadjiyeva, Nargiza S. Saidkasimova, Nargiza X. Otamuratova, Xolmamat N. Norboev

**Affiliations:** Tashkent State Medical University, Tashkent, Uzbekistan (B.B. Rakhimov, L.N. Tuychiev, N.T. Khamzayeva, N.U. Tadjiyeva, N.S. Saidkasimova, N.X. Otamuratova, X.N. Norboev); Republican Specialized Scientific-Practical Medical Center for Epidemiology, Microbiology, Infectious and Parasitic Diseases, Tashkent (N.U. Tadjiyeva, N.X. Otamuratova)

**Keywords:** meningococcal disease, *Neisseria meningitidis*, epidemic investigation, Central Asia, case fatality, lumbar puncture, post-exposure prophylaxis, surveillance, bacteria, meningitis/encephalitis, Tashkent, Uzbekistan

## Abstract

Tashkent, Uzbekistan, registered 290 invasive meningococcal disease cases during January–April 2026; the case fatality rate was 8.3%. Meningeal signs appeared in only 3.1% of patients and were the sole signs associated with death. *Neisseria meningitidis* serogroup surveillance and conjugate vaccine introduction will be needed to prevent invasive meningococcal disease in Uzbekistan.

Invasive meningococcal disease (IMD), caused by *Neisseria meningitidis*, can kill a healthy child within hours of symptom onset (*1*). Before the COVID-19 pandemic, IMD incidence had declined across most high-income countries owing to conjugate vaccination programs (*2*). After pandemic restrictions were lifted, IMD incidence rebounded. During 2023–2024, the United States recorded its highest annual total IMD cases since 2014 (*3*); Spain’s Aragon region reported a 27-fold case excess in early 2025 (*4*), and an outbreak struck Canterbury, England, in early 2026 (*5*). Uzbekistan is a lower-middle–income country within Central Asia (population ≈36 million). Uzbekistan has no published IMD epidemiology, and meningococcal conjugate vaccines are absent from the country’s national immunization schedule. During 2026, a total of 290 IMD cases were registered in Tashkent, Uzbekistan. We describe the clinical profile, laboratory findings, predictors of fatal outcome, and public health response to this IMD epidemic.

## The Study

We conducted a prospective epidemic investigation of all IMD cases registered at the Tashkent City Center for Sanitary-Epidemiologic Welfare and Public Health and applied 2018 World Health Organization case definitions (*6*). Confirmed cases required laboratory identification of *N. meningitidis* from a sterile site (blood culture or cerebrospinal fluid [CSF]). Probable cases met clinical criteria and had an epidemiologic link to a confirmed case, defined as household membership or attendance at the same institution within 10 days of primary IMD onset. Nasopharyngeal (NP) swab samples were collected from all patients during routine carriage screening according to the national protocol and were reported descriptively; they were not used to define case status. The ethics committee of Tashkent State Medical University approved this study (approval no. 20; June 12, 2026). Analysis of deidentified surveillance data did not require individual informed consent.

The 5 clinical signs, recorded as present or absent, were meningeal signs (nuchal rigidity, Kernig sign, or Brudzinski sign, documented by the admitting physician), loss of consciousness, hemorrhagic rash, fever, and vomiting. Clinical IMD severity was graded by the admitting physician as moderate, severe, fulminant, or extremely severe, following Uzbekistan’s national meningococcal disease management protocol. Cases with unknown outcomes at the data cutoff timepoint (April 14, 2026) were excluded from case-fatality calculations.

We performed statistical analyses by using SPSS Statistics 26 (IBM Corp., https://www.ibm.com). We compared continuous variables by using Welch’s *t*-tests. We tested binary associations between clinical signs and fatal outcomes by using a Pearson χ^2^ or Fisher exact test and calculated odds ratios (ORs) with 95% CIs. We used multivariable binary logistic regression with simultaneous entry of all covariates to model fatal outcomes. We assessed model calibration by using the Hosmer–Lemeshow test and discrimination by using area under the receiver operating characteristic curve.

A total of 290 IMD cases were registered in Tashkent during January 1–April 14, 2026 (Figure). Case numbers increased sharply through late January and February, peaked during February 12–18, and then declined through April, a pattern consistent with community-wide epidemic spread rather than a point-source outbreak.

**Figure:**
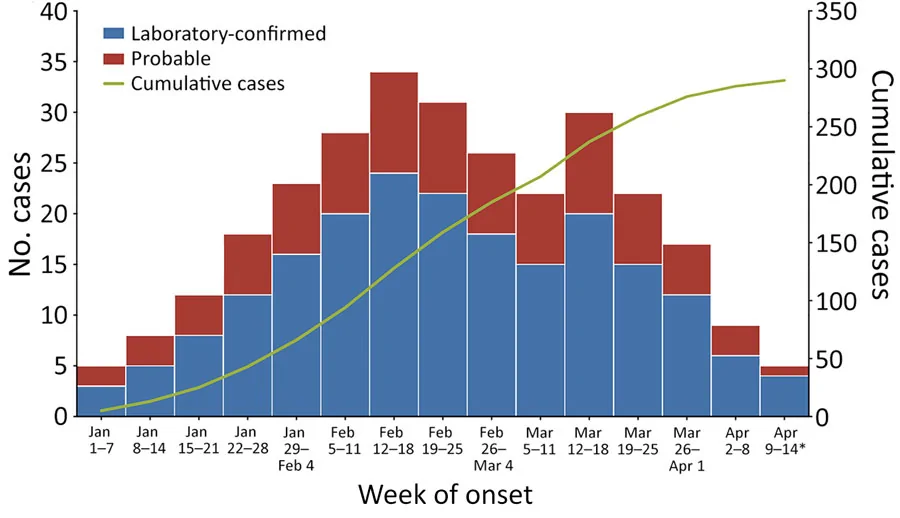
Weekly number of cases of invasive meningococcal disease in study of epidemic in Tashkent, Uzbekistan, January 1–April 14, 2026. Disease cases (n = 290) were registered in the invasive meningococcal disease case registry at the Tashkent City Center for Sanitary-Epidemiologic Welfare and Public Health. Cases were evaluated according to laboratory confirmation status and cumulative case counts. For laboratory-confirmed cases, *Neisseria meningitidis* was isolated from a sterile site (blood culture or cerebrospinal fluid). Probable cases met clinical criteria and had an epidemiologic link to a confirmed case. Asterisk indicates a partial week (April 9–14; 6 days).

Among 290 case-patients, 171 (59.0%) were male and 119 (41%) female (Table 1). Median patient age was 36 (interquartile range [IQR] 18–72) months. Children 2 to <5 years of age constituted the largest (41.0%) group, followed by 5 to <10 years (17.6%), 1 to <2 years (14.5%), and <1 year of age (5.9%). Cases were distributed across all 12 Tashkent administrative districts (Appendix Table 1). The highest incidence was observed in Olmazor (37 cases). Fifteen cases were reported from other regions of Uzbekistan.

**Table 1:** Demographic and clinical characteristics of patients in study of invasive meningococcal disease epidemic in Tashkent, Uzbekistan, January 1–April 14, 2026*

Characteristic	Patients
Total	290 (100)
Sex	
M	171 (59.0)
F	119 (41.0)
Age at onset, mo., median (IQR)	36 (18–72)
Age group, y	
<1	17 (5.9)
1 to <2	42 (14.5)
2 to <5	119 (41.0)
5 to <10	51 (17.6)
10 to <18	13 (4.5)
>18	6 (2.1)
Missing	42 (14.5)
Outcome, n = 261 with known outcome†	
Discharged alive	237 (90.8)
Death, CFR 8.3%‡	24 (9.2)
Clinical sign at admission, n = 261†	
Fever >38°C	165 (63.2)
Hemorrhagic rash	165 (63.2)
Loss of consciousness	162 (62.1)
Vomiting	161 (61.7)
Meningeal signs§	8 (3.1)
Laboratory test results for sterile site	
Blood culture positive	176 (60.7)
CSF culture positive, 56 LPs performed	28 (9.7)
Sterile site confirmed, blood or CSF	187 (64.5)
Nasopharyngeal carriage screening positive¶	164 (56.6)
CSF not obtained	234 (80.7)

*Values are no. (%) except as indicated. CFR, case fatality rate; CSF, cerebrospinal fluid; IQR, interquartile range; LP, lumbar puncture. †Twenty-nine cases were excluded because outcome was unknown at the data cutoff timepoint. Data for subgroups are no. (%) of 261 cases with known outcome. ‡CFR = deaths/all 290 cases × 100. §Meningeal signs were defined as the presence of nuchal rigidity, Kernig sign, or Brudzinski sign (any one of which was sufficient as a clinical sign). ¶Nasopharyngeal swab samples taken from all patients as routine carriage screening; not used to define case status.

Outcome data were available for 261 (90.0%) patients; 29 (10.0%) cases remained unresolved at the cutoff timepoint. Among those 261 patients, 237 (90.8%) were discharged alive and 24 (9.2%) died, yielding an overall case-fatality rate (CFR) of 8.3% (24/290). The median age was 48.0 (IQR 24–120) months for patients who died versus 33.0 (IQR 16–60) months for survivors (Welch’s *t*-test p<0.001).

Fever and hemorrhagic rash were each documented in 165 (63.2%) cases, loss of consciousness in 162 (62.1%) cases, and vomiting in 161 (61.7%) cases. Meningeal signs were observed in 8/261 (3.1%) cases having known outcomes. Univariable analysis showed meningeal signs were the sole clinical sign significantly associated with death. CFR was 50.0% among case-patients with meningeal signs versus 7.9% in those without (OR 11.65 [95% CI 2.71–50.13]; Fisher exact test p = 0.003) (Table 2). The 4 high-prevalence signs showed no significant association with fatal outcome (all p>0.50) (Table 2).

**Table 2:** Clinical predictors of fatal outcome determined by univariable and multivariable analyses in study of invasive meningococcal disease epidemic in Tashkent, Uzbekistan, 2026*

Variable	CFR present, %	CFR absent, %	Crude OR (95% CI)	Adjusted OR (95% CI)	p value
Clinical signs, univariable					
Meningeal signs	50.0	7.9	11.65 (2.71–50.13)	NA	0.003†
Loss of consciousness	8.8	9.8	0.89 (0.38–2.08)	NA	0.785
Hemorrhagic rash	9.3	9.1	1.02 (0.43–2.43)	NA	0.964
Fever >38°C	9.3	9.1	1.02 (0.43–2.43)	NA	0.964
Vomiting	8.2	10.7	0.75 (0.32–1.75)	NA	0.503
Multivariable logistic regression‡					
Age at onset, 12 mo.				1.06 (1.02–1.11)	0.005
Sterile site confirmed				1.61 (0.50–5.15)	0.424

*N = 261 case-patients. CFR, case fatality rate; NA, not applicable; OR, odds ratio. †Fisher exact test; all other p values determined by Pearson χ^2^ test. ‡Model included age at onset, sterile-site laboratory confirmation, and all 5 clinical signs. Binary signs did not converge owing to quasi-complete separation caused by the extreme rarity of meningeal signs (n = 8; 3.1%) and are not shown as adjusted OR estimates.

Multivariable logistic regression identified older age at onset as an independent predictor of death (adjusted OR per 12 months of age 1.06 [95% CI 1.02–1.11]; p = 0.005) (Table 2). The model displayed acceptable discrimination (area under the curve 0.712 [95% CI 0.595–0.828]; p = 0.001), calibration (Hosmer–Lemeshow test p = 0.98), and performance (Nagelkerke R^2^ = 0.193). Binary clinical signs did not yield stable multivariable estimates because of few meningeal signs (n = 8).

In Tashkent, all persons with suspected IMD are admitted to City Children’s Infectious Disease Hospital No. 1; specimen collection is differentiated according to clinical disease form. Patients manifesting meningeal signs undergo lumbar puncture within the admissions area; those with meningococcemia without meningeal signs have blood drawn for culture; and those with mixed-form disease have CSF, blood culture, and NP swab samples collected. CSF was not obtained in 234 (80.7%) cases, reflecting predominance of meningococcemia and mixed-form disease rather than deviation from established protocol.

Among 56 patients who had lumbar punctures, 28 (50.0% of specimens; 9.7% of all cases) had confirmed *N. meningitidis* in CSF. Blood culture confirmed *N. meningitidis* in 176 (60.7%) patients. Sterile-site laboratory confirmation (blood culture or CSF) was achieved in 187/290 (64.5%) cases. NP carriage screening results were positive for *N. meningitidis* in 164 (56.6%) patients; other organisms were identified in 8 (2.8%) NP swab samples. Sterile-site laboratory confirmation was not associated with fatal outcome (CFR 8.4% in confirmed vs. 8.1% in unconfirmed cases; p = 0.94) (Appendix Table 2).

Postexposure ring vaccination was documented for contacts of 103 (35.5%) index patients; chemoprophylaxis was reported for contacts of 20 (6.9%) index patients. Approximately 8,024 vaccine doses were deployed citywide, and 4,862 index patient contacts were vaccinated under emergency authorization.

## Conclusions

Tashkent’s 2026 IMD epidemic occurred during a global post-COVID-19–pandemic meningococcal resurgence, as immunologic debt accumulated during years of restricted social mixing (*7*,*8*). A CFR of 8.3% in Tashkent falls within the globally reported range for settings without optimized intensive-care protocols (*9*,*10*). The clinical profile was markedly atypical; meningeal signs, documented in 40%–70% of patients in case series from Europe (*11*,*12*), were observed in <3.1% of cases in Tashkent, reflecting dominance of meningococcemia and mixed-form disease. The low number of patients with meningeal signs is phenotypically consistent with serogroup C or W strains, which characteristically produce fulminant bacteremia and minimal meningeal inflammation (*13*). However, serogroup confirmation was not achieved during the epidemic.

The near absence of meningeal signs carries direct clinical implications. Caregivers and clinicians are trained to identify IMD primarily by neck stiffness and photophobia; thus, they might not recognize IMD when it manifests as hemorrhagic septicemia alongside altered consciousness (*14*).

The first limitation of our study is that serogroup and antimicrobial drug susceptibility data were unavailable for the entire cohort. Second, outcome data were missing for 10% of cases, and disease severity grading was inconsistently recorded. Finally, ring vaccination reached documented contacts of only 35.5% of index patients and chemoprophylaxis reached <7% of, both well below World Health Organization postexposure recommendations (*15*).

In summary, Tashkent’s 2026 surge in IMD cases indicates a substantial, underrecognized public health burden. Serogroup surveillance and conjugate vaccine introduction will be needed to prevent IMD in Uzbekistan.

Appendix Additional information for clinical characterization of invasive meningococcal disease epidemic, Uzbekistan, 2026.
