# Supplementary material for: Clinical Characterization of Invasive Meningococcal Disease Epidemic, Uzbekistan, 2026
Source: Emerg Infect Dis. 2026 Oct;32(10):1712–5. doi: 10.3201/eid3210.261144 (PMC13632880; doi:10.3201/eid3210.261144)
Supplement: Supplementary file 1 [file 26-1144-Techapp-s1.pdf]

Article DOI: https://doi.org/10.3201/eid3210.261144
EID cannot ensure accessibility for supplementary materials supplied by authors. Readers who have difficulty accessing supplementary content should contact the authors for assistance.

Clinical Characterization of Invasive Meningococcal Disease Epidemic, Uzbekistan, 2026

Appendix

Appendix Table 1 shows district-level case counts and approximate attack rates, calculated by using 2024 intercensal population estimates from the Tashkent City Statistical Department. Population figures carry inherent uncertainty attributable to the interval between censuses (2021 and anticipated 2026), particularly for districts undergoing rapid residential development (Sergeli, Yangi Hayot). Attack rates are reported for the 15-week epidemic period (January 1–April 14, 2026) and should not be annualized.

Appendix Table 2 shows overlap in laboratory specimen positivity across 3 specimen types. The number of cases with both blood culture and cerebrospinal fluid (CSF) positive samples was derived arithmetically: n(both) = n(blood positive) + n(CSF positive) − n(sterile-site confirmed by either). Cases with positive nasopharyngeal swab samples, but without sterile-site confirmation (n = 14), were reclassified as probable cases in the revised manuscript, consistent with WHO 2018 case definitions requiring laboratory identification of *Neisseria meningitidis* from a normally sterile site for confirmed status.

Appendix Table 1. District-level case counts and approximate attack rates for invasive meningococcal disease, Tashkent, Uzbekistan, January 1–April 14, 2026*

District†	No. cases	Estimated population, × 10^3‡	Approximate attack rate per 100,000 persons§	% Documented ring vaccination¶
Olmazor	37	320	11.6	35.5
Yunusobod	30	390	7.7	40.0
Chilonzor	29	370	7.8	34.5
Shaykhontohur	28	350	8.0	32.1
Mirzo Ulugbek	26	310	8.4	42.3
Uchtepa	24	295	8.1	29.2
Yashnobod	22	280	7.9	36.4
Mirabad	20	260	7.7	35.0
Yakkasaroy	18	240	7.5	33.3
Yangi Hayot	17	230	7.4	29.4
Sergeli	16	270	5.9	31.3
Bektemir	8	185	4.3	25.0
Subtotal, 12 districts	275	3,500	7.8 (range 4.3–11.6)	35.3
Other regions of Uzbekistan	15	NA	NA	NA
Total	290	NA	NA	35.5 (103/290)

*NA, not applicable.
†Districts are listed in descending order of case count.
‡2024 intercensal population estimates from the Tashkent City Statistical Department. Estimates carry uncertainty owing to rapid residential development in some districts.
§Attack rates calculated for the 15-week epidemic period (January 1–April 14, 2026) only; not annualized.
¶Ring vaccination was documented for contacts of index cases according to the surveillance registry. Coverage might be underestimated if vaccination was administered but not recorded.

Appendix Table 2. Laboratory specimen positivity combinations among 290 invasive meningococcal disease cases, Tashkent, Uzbekistan, 2026*

Specimen combinations	No. (%) samples†
Sterile-site specimen results	
Blood culture positive and CSF positive (both)‡	17 (5.9)
Blood culture positive only (CSF negative or not taken)	159 (54.8)
CSF positive only (blood culture negative or not taken)	11 (3.8)
Sterile site confirmed (blood culture or CSF; any)	187 (64.5)
No sterile-site confirmation (neither blood nor CSF positive)	103 (35.5)
Nasopharyngeal carriage screening§	
NP swab positive and sterile-site confirmed	150 (51.7)
NP swab positive without sterile-site confirmation¶	14 (4.8)
NP swab negative or not taken	126 (43.4)
Total positive NP swabs	164 (56.6)

*CSF, cerebrospinal fluid; NP, nasopharyngeal.
†Total no. specimens = 290.
‡Numbers were derived using the equation: n(both blood and CSF positive) = n(blood+) + n(CSF+) − n(sterile-site confirmed) = 176 + 28 – 187 = 17.
§Nasopharyngeal swab samples were obtained from all admitted patients as routine *Neisseria meningitidis* and *Streptococcus pneumoniae* carriage screening per national protocol. They are classified here as carriage screening data but not used to define case confirmation status.
¶These 14 cases, previously classified as confirmed in an earlier version of this manuscript based on NP swab samples alone, have been reclassified as probable cases in the revised submission, consistent with WHO 2018 case definitions requiring sterile-site specimen confirmation.
